# Estimation of the aboveground biomass and carbon stocks in open Brazilian Savannah developed on sandy soils

**DOI:** 10.1186/s13021-019-0121-0

**Published:** 2019-05-04

**Authors:** Camila Paula de Oliveira, Márcio Rocha Francelino, Mayara Daher, Emanuel José Gomes de Araújo, Leonardo de Souza Sanches, Kauanna Domingues Cabral de Andrade, Júlia Santos Nunes de Campos

**Affiliations:** 1 Departamento de Silvicultura, Instituto de Florestas, Universidade Federal Rural do Rio de Janeiro, BR 465 km 7, Seropédica, 23890-000 Brazil; 2 Pós-Graduação em Ciências Ambientais e Florestais, Instituto de Florestas, Universidade Federal Rural do Rio de Janeiro, BR 465 km 7, Seropédica, 23890-000 Brazil; 3 Departamento de Solos, Universidade Federal de Viçosa, Av. PH Rolfs, s/n, Viçosa, 36570-900 Brazil

**Keywords:** Biomass models, Sand soil, Allometric equations, Savannic formations

## Abstract

**Background:**

The Cerrado is the second largest biome in Brazil and the most biodiverse tropical savannah in the world and acts as a great sequester of atmospheric carbon. The lack of studies related to the quantification of its total biomass compromises the understanding of the dynamics of CO_2_ in this biome. Thus, it is relevant to develop studies aiming at obtaining accurate estimates of the carbon stock in the different phytophysiognomies that make the Cerrado, to include them in appropriate forest management models. Based on the hypothesis that the amount of carbon stored can vary according to the vegetation typology and vegetation compartments, the aerial stock of dry biomass and carbon were estimated in different compartments (arboreal, herbaceous-shrub and litter). The study was developed in open Brazilian savannah and soils on the sandstone and discussed the effect of fire on this phytophysiognomy. For the arboreal compartment were adjusted mathematical models to fit the biomass equations to estimate the individual stock of the trees in this compartment. The results of the stocks were discussed considering the effect of fire on the phytophysiognomy.

**Results:**

Based on the precision and extra distribution measures, the Schumacher-Hall (non-logarithmic) equation presented better results to estimate the individual biomass and carbon stocks of the open Brazilian savannah trees. The aboveground biomass was 12.88 Mg ha^−1^, corresponding to a total carbon stock of 5.91 Mg ha^−1^, where most of the stocks are in the herbaceous-shrub compartment (44%). The arboreal compartment accounts for the smallest part of the stocks, followed by the litter.

**Conclusions:**

The observed values are in the interval verified for other areas of savannah studied in Brazil. The values verified for the open Brazilian savannah in sandy soils are at the lower limit of this range, due to the nutrient-poor nature of this type of soil. The distribution of stocks in the different compartments above the ground points to the fragility of this environment to the random fire effect, common in the region. That shows the need for conservation measures for vegetation maintenance and soil protection to preserve adequate nutrient cycling in the ecosystem.

## Background

Topics that seek to understand the causes and effects of global climate change, as well as the decision-making necessary for the proper management of natural resources, permeate the current scientific discussions. Above all, because the increase in concentrations of atmospheric carbon dioxide and its direct effect on climate variability leads to uncertainties about its influence on the quality of human life on the planet [11]. In Brazil, the conversion of forested areas to other forms of land use accounts for about 77% of carbon dioxide emissions in the atmosphere [4]. This data demonstrates the need to know and conserve remaining forest ecosystems, which, besides providing this environmental service, offer other indispensable ones. So, there are few studies on carbon stock and dynamics in areas of Cerrado [28, 29].

In the case of the Cerrado biome, the change in land use intensified in the 1970s due to the expansion of the agricultural frontier in this biome, which presents favorable relief and soil conditions for mechanization processes of agricultural production, besides low land prices [3, 13]. Still, according to these authors, the deforestation also occurred in an unsustainable way for the production of coal. In this context, in the last 40 years, about 50% of the Cerrado biome has been completely deforested and converted to fullanthropic uses [5]. The degradation of this biome occurred more intensely in the western region of Bahia state, especially in the São Francisco River basin, being this the region that more evolves on the conversion of vegetated areas in the biome [5].

The Cerrado is the second largest biome in Brazil and the most biodiverse tropical savannah in the world [19], it acts as a great sequester of atmospheric carbon [14, 15, 34]. The lack of studies related to the quantification of its total biomass compromises the understanding of the dynamics of CO_2_ in this biome [28]. Thus, it is relevant to develop studies aiming at obtaining accurate estimates of the carbon stock in the different phytophysiognomies that compose the Cerrado, to include them in appropriate forest management models for this biome considering its different phytophysiognomies [31, 33].

Based on the hypothesis that the amount of carbon stored can vary according to the vegetation typology, this work had the aim to quantify the total above ground stocks of dry biomass and carbon for the arboreal, herbaceous-shrub and litter compartments in an open Brazilian savannah (OBS) phytophysiognomy of the Cerrado biome with occurrence in soils of medium to the sandy texture from the sandstone.

## Methods

### Study area

**Fig. 1:**
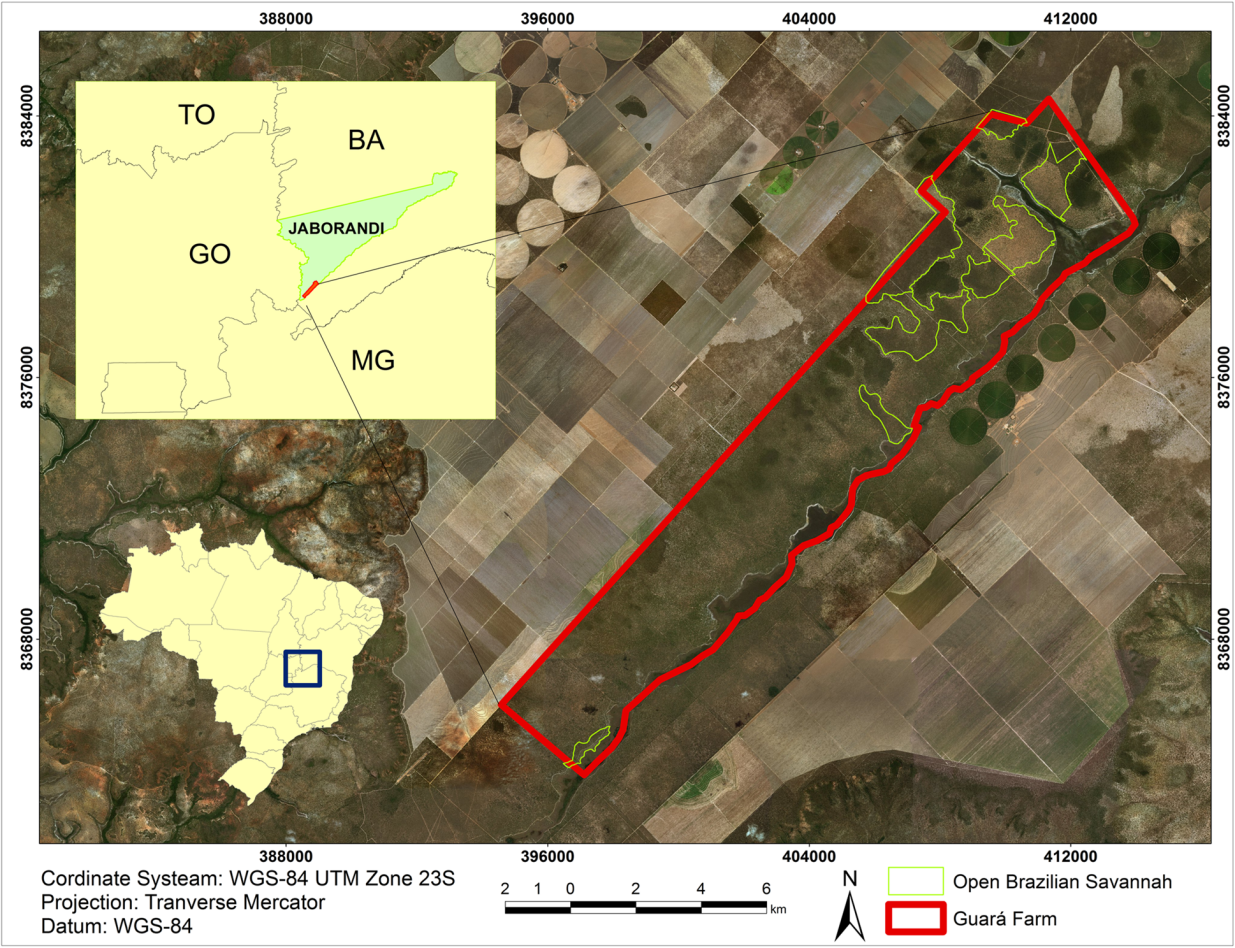
Location of study area, boundaries of the states of Bahia, Minas Gerais and Goiás

The study was developed in a continuous area of the Cerrado biome of approximately 9570 ha in the central region of Brazil (boundaries of the states of Bahia, Minas Gerais, and Goiás) (Fig. 1). The regional climate is Aw, according to the classification of Köppen, characterized a tropical dry winter, presenting a mean annual temperature of 24 °C and mean annual rainfall of 870 mm [9]. The altitude is approximately 850 m, and the relief is flat.

The area is located in the region of occurrence of the plateau that forms the Urucuia Formation [8], in this lithological substrate, quartz sandstones of varied colors is predominant, whose granulometry varies from thin to medium. The developed soils of these sandstones present a sandy loam texture, dominated by Oxisols and Quartzipsamments [35].

The area is inserted in the Cerrado biome, being almost covered by native vegetation, with a great diversity of environments, including different phytophysiognomies of the savannahs [25]. The OBS, a phytophysiognomy chosen for the study, covers about 1022 ha, which represents 10.7% of the total area of this continuum biome.

The study was conducted in an area < 30 ha covered by open Brazilian savannah. The area was defined based on the real representativeness of the phytophysiognomy and not being designated as a Conservation Unit, Legal Reserve or Permanent Preservation Area, making it possible to get authorization from the competent environmental agency for the suppression of trees, which were used for the destructive sampling of biomass quantification. In this area of 30 ha, the forest inventory was first made to know the floristic composition and the structure of the tree component of the open Brazilian savannah. For this, 20 plots of 1000 m^2^ (20 m × 50 m) were allocated randomly in the research area. In each of the plots were collected the data of diameter and a total height of the trees that met the criterion of inclusion of the diameter measured at 30 cm from the soil (d_30cm_) equal to or greater than 5 cm (d_30cm_ ≥ 5 cm), as recommended by Felfili et al. [12]. The trees measured had the botanical material collected to identify the botanical species present.

### Sampling of the arboreal, herbaceous-shrub and litter compartment

The biomass stored in the studied physiognomy was quantified by vegetation compartment (arboreal, herbaceous-shrub, and litter). For the sampling of the arboreal compartment were considered the woody individuals (trees) with a diameter at 30 cm from the ground level (d_30cm_) ≥ 5 cm. According to data from the forest inventory carried out in the area, 60 trees were chosen to compose the sample of trees that were cut for quantification of the biomass by a destructive method for later adjustment of the biomass equations. The selection of these trees was made according to the diametric structure of the studied open Brazilian savannah and the species composition, being sampled those of higher importance value index (IVI), as presented in Oliveira [24]. Later they were cut to 30 cm of the ground, and the total height of the tree was measured. Once the diametric measurements were taken, its components (leaves, thin branches, thick branches, trunk, flowers, and fruits) were weighed. After weighing, samples of each component were collected and conditioned in plastic bags to get the weight of dry matter in the laboratory. From the trunks, disks were removed at the 0.30 m positions, at half the total height, and at the total height, whose samples were dried in a forced circulation oven, with a temperature around 70 °C, until obtaining a constant mass.

From the data obtained from each tree (weight), statistical models were adjusted for the estimation of dry biomass per tree (non-destructive), as a function of the variables diameter (d_30cm_) and total height (ht), as well as their combinations. For this, different linear and non-linear models were tested according to Scolforo et al. [37].

The linear models were adjusted using regression analysis with the ordinary least squares method to estimate the parameters [36]. The non-linear models were adjusted by iterations. The data were tested for the assumptions of normality and homogeneity, using the Shapiro–Wilk test (α = 0.05) and f-test (α = 0.05). Graphical analysis was performed to verify the correlation between the dependent and independent variables. The choice of the best model was based on the adjusted coefficient of determination (R^2^_adjust._), on the significance of the coefficients at 95% probability, on the standard error of estimate in percentage terms (Sxy%), and the graphical distribution of the residues. For the models with the transformed dependent variable, the residual standard error was corrected in the original scale of the dependent variable, according to Scolforo [36]. The analysis of the difference between the values observed and those estimated by the chosen equation was performed by paired t-test (α = 0.05).

For the herbaceous-shrub compartment sampling, woody individuals with d_30cm_ < 5 cm and total ht < 1 m, and undergrowth, including small herbs, grasses, and palms (*Syagrus petrea*) were distributed in the area. Sampling for quantification of the biomass in this compartment was performed using 20 fixed sub-plots of 4 m^2^ (2 m × 2 m). The 4 m^2^ subplots were allocated in the center of the largest parcels of forest inventory (20 m × 50 m). In each of the 20 subplots, all the wet material was separated into the components and weighed (leaves, twigs and grasses, branches and grasses). Samples of each component were taken to get moisture contents and dry matter weight in the laboratory. A sampling of the biomass present in the litter was carried out by 20 fixed subplots of 1 m^2^ (1 m × 1 m). These subplots were also allocated within the larger parcels of the forest inventory. The procedure was to allocate the subplot in one of the four corners of the largest part of the forest inventory. In each of the 20 subplots, all the wet material present was weighed, and one sample was collected to get moisture content and dry matter weight in the laboratory.

### Estimation of stocks of dry biomass and carbon

For the herbaceous-shrub and litter compartments, the biomass values obtained in the field were converted into dry biomass quantity per hectare from the moisture content determined in the laboratory. The chosen biomass equation was applied to the data collected in the forest inventory to estimate the dry biomass stock of the arboreal compartment. This allowed estimating the stocks per hectare, as well by individuals, species and diametric classes of the Cerrado compartment. Estimates for each compartment were made by confidence interval at 95% probability (α = 0.05). For the arboreal and herbaceous-shrub compartments, the total dry biomass was converted to carbon considering the proportion of 47% [18]. For the litter, the carbon concentration was considered 44.36% [22].

## Results and discussion

### Adjustment of models to estimate dry biomass and carbon stocks of individual trees

The sample for quantification of the biomass contained in the arboreal compartment of the studied physiognomy, OBS, counted with 60 trees, distributed in five diametric classes (Table 1) and among eight different species: *Kielmeyera coriacea (17), Pouteria ramiflora (15), Kielmeyera petiolaris (8), Hancornia speciosa (7), Pouteria torta (5), Eugenia dysenterica (2), Vochysia tucanorum (3), Palicourea rigida (3).*

**Table 1:** Frequency of arboreal individuals sampled by diametric class in the OBS for quantification of biomass by a direct method

Classes of d_30cm_	Absolute frequency	Relative frequency (%)	Frequency of trees sampled
5.0–8.9	829	74.62	23
9.0–12.9	195	17.55	17
13.0–16.9	59	5.31	13
17.0–20.9	17	1.42	5
> 20.9	11	0.87	2
Total	1.111	100	60

All models adjusted were significant (p < 0.05) (Table 2). The R^2^_adjust._ ranged from 71.20 to 94.55%, which represents a high correlation between the dependent variables (biomass and independent variables (d_30cm_ and ht).

**Table 2:** Estimation of the parameters and precision measurements of the adjusted equations for the estimation of the dry biomass stock (kg) as a function of the d_30cm_ in the open Brazilian savannah

Model	*β*_0_	*β*_1_	*β*_2_	*β*_3_	R^2^ % (adjust.)	Sxy (kg)	Sxy (%)
Hohenald Krenm	7.7068*	− 2.4160*	0.2447	–	71.20	11.13	70.13
Brenac	− 1.8494*	2.0488	− 7.8893*	–	89.46	11.58	72.89
Spurr	− 2.8828*	0.0352	–	–	89.04	6.87	43.26
Schumacher-Hall (logarithmic)	− 4.32	2.0157	1.5258	–	94.19	5.72	35.99
Stoate (Australian)	4.1867*	− 0.1461	0.0649	− 0.6371*	93.87	5.14	32.34
Spurr (logarithmic)	− 4.446	1.1305	–	–	93.95	6.79	42.74
Honner	− 5.5889	57.2571	–	–	94.50	4.87	30.65
Takata	40.3464	− 0.5224	–	–	88.67	6.98	43.98
Schumacher-Hall	0.01313	1.7793	2.0228	–	94.55	4.85	30.50

*Non-significant coefficients (p > 0.05)

The Sxy% varied from 30.50 to 72.89%, demonstrating that the pattern of high heterogeneity of Cerrado samples [10, 31, 38], which is confirmed by the dendrometric characteristics and the stocks per plant compartment of the trees sampled in the OBS to adjust of the models (Table 3). In this table it is observed that the Schumacher-Hall model (non-logarithmic) presented the best measures of precision and also satisfactory residual distribution, indicating trend-free estimates. Thus, it is the model chosen to estimate the individual biomass and, so carbon stocks, of the studied phytophysiognomy trees, since there was no significant difference between the values observed and estimated by the equation when compared by the paired t-test (α = 0.05):

**Table 3:** Minimum and maximum values, mean, standard deviation, and coefficient of variation relative to the dendrometric characteristics and different stocks per compartment of the trees sampled for the adjustment of the models in the OBS

Variáveis	Minimum	Maximum	Mean	Standard deviation	CV (%)
d_30_ (cm)	5.09	23.55	11.11	4.48	40.42
h (m)	1.75	6.2	3.2	0.92	28.75
Weight—leaves (kg)	0.06	6.39	1.148	1.557	135.96
Weight—thin branches (kg)	0.18	26.344	3.609	4.376	121.38
Weight—thick branches (kg)	0.135	41.745	6.512	9.037	138.72
Weight—trunk (kg)	1.021	119.32	16.584	21.411	129.13
Weight—fruits (kg)	0.22	3.087	1.203	1.632	135.49
Weight—total (kg)	1.777	193.574	27.703	34.618	124.93
Total dry biomass (kg)	0.883	124.564	15.882	20.755	130.66
Total carbon (kg)	0.415	58.545	7.465	9.755	130.68

$${\text{Dry biomass }}\left( {\text{kg}}\right): {\text{y}} = \, 0.0 1 3 1 3 {{\text{d}}_{{ 30{\text{cm}}}}}^{{ 1, 7 7 9 3}}\, {\text{h}}^{ 2,0 2 2 8}$$

### Biomass and carbon stocks in the arboreal compartment

The biomass stock in the arboreal compartment was 3.5 Mg ha^−1^ ([2.4 Mg ha^−1^ ≤ μ ≤ 4.6 Mg ha^−1^] = 0.95), corresponding to a stock carbon mean of 1.7 Mg ha^−1^ ([1.1 Mg ha^−1^ ≤ μ ≤ 2.2 Mg ha^−1^] = 0.95) in this compartment (Table 4). In nine localities of OBS, Ottmar et al. [27] verified dry biomass stock ranging from 3.31 to 29.84 t ha^−1^. The lowest value (3.31 t ha^−1^) was observed in an OBS sampled in the Grande Sertão Veredas National Park area (close to the area of this study), corroborating with the result found in this study, which presents a mean of 3.5 t ha^−1^ of dry biomass stored in its arboreal compartment. According to Castro and Kauffmam [7], total aboveground biomass (including trees, shrubs, undergrowth, and litter) tends to be smaller as vegetation tends to be less dense. Thus, for this same area, Oliveira et al. [26] found values of dry biomass of 10.9 Mg ha^−1^, for the arboreal component in the *typical* savannah.

**Table 4:** Dry biomass and carbon stocks, density and dominance of individuals in the arboreal compartment of the OBS

Variable	Mean	S_ӯ_t	(S_ӯ_t) %	Inferior limit	Superior limit
Density (ind. ha^−1^)	556	95	17.04	461	650
Basal area (m^2^ ha^−1^)	3.5	0.55	15.9	2.9	4.0
Biomass (t ha^−1^)	3.5	1.1	31.19	2.4	4.6
Carbon (t ha^−1^)	1.6	0.51	31.19	1.1	2.2

S_ӯ_t: absolute sample error; (S_ӯ_t) %: Relative sample error; α: 0.05

The sampling error for the biomass and carbon variables in the studied physiognomy was higher than 20% (Table 4), indicating that for more efficient sampling of these variables it is necessary to allocate more plots in the field. The high variability and the high sampling error were due to the presence of a plot in which 108 individuals were sampled, giving density much higher than the mean density calculated per plot, which was 55 ind. 0.1 ha^−1^ on this physiognomy.

About the contribution of species, it was observed that four species accounted for more than 50% of biomass and carbon stocks, being *Kielmeyera coriaceae, H. speciosa, P. ramiflora,* and *K. petiolaris*, which were also responsible for the higher IVI in this savannah (Table 5). *Salvertia convallareiodora,* represented by only three individuals inventoried presented significant stocks due to the presence of a large individual, responsible for the largest diameter recorded.

**Table 5:** Distribution of dry biomass (Mg ha^−1^) by species and diametric classes in OBS

Species	Diametric classes (cm)						∑
	5–8.9	9–12.9	13–16.9	17–20.9	21–24.9	> 25	
*Kielmeyera coriaceae* ^a^	0.235	0.255	0.085	0.022	–	–	0.598
*Hancornia speciosa* ^a^	0.085	0.174	0.051	0.054	0.074	–	0.438
*Pouteria ramiflora* ^a^	0.223	0.175	0.039	–	–	–	0.437
*Kielmeyera petiolaris* ^a^	0.185	0.099	0.073	0.003	0.041	–	0.401
*Salvertia convallariaedora*	0.003	–	–	–	–	0.369	0.372
*Pouteria torta*	0.043	0.044	0.032	0.021	0.072	0.130	0.341
*Eugenia dysenterica*	0.048	0.1	0.026	0.106	–	–	0.281
*Tabebuia aurea*	0.017	0.037	0.074	–	–	–	0.129
*Vochysia tucanorum*	0.003	0.039	–	0.084	–	–	0.125
*Macherium opacum*	–	–	–	0.062	–	–	0.062
*Mouriri elliptica*	0.005	0.005	–	0.049	–	–	0.059
*Hymenaea stigonocarpa*	0.001	–	–	–	0.052	–	0.053
*Andira vemifuga*	0.005	0.005	0.023	–	–	–	0.033
*Enterolobium gummiferum*	0.002	0.006	–	0.019	–	–	0.027
*Handroanthus ochraceus*	0.000	–	0.016	–	–	–	0.016
*Palicourea rigida*	0.01	0.004	0.002	–	–	–	0.016
*Qualea parviflora*	–	0.015	–	–	–	–	0.015
*Annona crassiflora*	0.007	0.005	–	–	–	–	0.012
*Agonandra brasiliensis*	0.002	0.009	–	–	–	–	0.011
*Dimorphandra mollis*	–	–	0.011	–	–	–	0.011
*Byrsonima verbascifolia*	0.002	0.005	–	–	–	–	0.007
*Acosmium* sp.	–	0.007	–	–	–	–	0.007
*Aspidospermas tomentosum*	–	0.006	–	–	–	–	0.006
*Erythroxylum suberosum*	0.005	0.001	–	–	–	–	0.006
*Tocoyena formosa*	0.004	–	–	–	–	–	0.004
*Lafoensia pacari*	0.002	–	–	–	–	–	0.002
*Myrcia guianensis*	0.002	–	–	–	–	–	0.002
*Byrsonima* sp.2	–	0.001	–	–	–	–	0.001
*Bowdichia virgilioides*	0.001	–	–	–	–	–	0.001
*Byrsonima* sp.1	0.001	–	–	–	–	–	0.001
*Eugenia* sp1.	0.001	–	–	–	–	–	0.001
*Psidium pohlianum*	0.001	–	–	–	–	–	0.001
*Rourea induta*	0.001	–	–	–	–	–	0.001
*Connarus suberosus*	0.000	–	–	–	–	–	0.000
∑	0.896	0.993	0.431	0.420	0.239	0.499	3.477
% of total	25.76	28.56	12.40	12.08	6.86	14.34	100.00

^a^Higher importance value index (IVI) in open Brazilian savannah

By relating the biomass production and, so carbon, with the diametric structure of the communities, there was an increase from the first to the second class, from which there was a decrease to the penultimate class. The first three classes account for more than 60% of the biomass and carbon stored in the arboreal component, with the most stock in the second diametric class, presenting 0.9 t ha^−1^, which corresponds to 28.55% of the total stock of this compartment in this physiognomy.

Although in the higher diametric classes are the largest individual stock, the high number of individuals in the lower classes makes the stocks per hectare higher in this range, revealing that small individuals are an important part for the maintenance of biomass and carbon stocks, as well as the functioning of this ecosystem.

### Dry biomass and carbon stocks of the herbaceous-shrub component

The dry biomass mean stock was 5.63 Mg ha^−1^ ([5.00 Mg ha^−1^ ≤ µ ≤ 6.28 Mg ha^−1^] = 0.95), corresponding to a mean stock of carbon of 2.64 Mg ha^1^ ([2.35 Mg ha^−1^ ≤ µ ≤ 2.95 Mg ha^−1^] = 0.95). About the stocks by components of the compartment studied, grasses account for about 96% of the dry biomass and carbon stocks (Table 6).

**Table 6:** Dry biomass and carbon stocks in the different components of the herbaceous-shrub compartment of OBS

Variable	Mean	S_ӯ_t	(S_ӯ_t) %	Inferior limit	Superior limit	% of Total
Biomass—total (Mg ha^−1^)	5.63	0.64	11.39	5.00	6.28	100
Biomass—grasses (Mg ha^−1^)	5.42	0.57	10.48	4.85	5.98	96.28
Biomass—branches (kg ha^−1^)	17.67	15.05	85.15	2.62	32.73	0.31
Biomass—leaves (kg ha^−1^)	4.74	3.61	76.30	1.12	8.35	0.08

S_ӯ_t: absolute sample error; (S_ӯ_t) %: relative sample error; α: 0.05

Ottmar et al. [27] observed biomass of grasses varying from 1.76 to 9.95 Mg ha^−1^ for nine different locations studied in the OBS, while for typical Brazilian savannah the values ranged from 1.11 to 1.69 Mg ha^−1^. According to Ribeiro and Walter [32], the OBS represents the lowest and least dense form of the typical Brazilian savannah, where the shrub and herb layer is the most prominent when compared to the dense and typical subtypes, especially the grassy cover.

In the study area, the presence of woody shrubs is almost absent, the grassy layer is predominant, where *Syagrus petraea* individuals are widely distributed. The presence of natural regeneration individuals also occurred in the sampled plots, with individuals of *Kielmeyera coreaceae, P. ramiflora, Connarus suberosus,* and *Palicourea rígida*. According to Silva [39], the grassy layer is characterized by the presence of *Echinolaena inflexa*, *Paspalum gardnerianum*, *Paspalum polyphyllum,* and *Eragrostis* sp.

### Dry biomass and carbon stocks of the litter

In the studied physiognomy a dry biomass mean of 3.75 Mg ha^−1^ ([2.80 Mg ha^−1^ ≤ µ ≤ 4.70 Mg ha^−1^] = 0.95) was stored in the litter, which corresponds to a carbon stock mean of 1.66 Mg ha^−1^ ([1.24 Mg ha^−1^ ≤ μ ≤ 2.08 Mg ha^−1^] = 0.95) in this compartment (Table 7).

**Table 7:** Dry biomass and carbon stocks present in the litter of OBS

Variable	Mean	S_ӯ_t	(S_ӯ_t) %	Inferior limit	Superior limit
Dry biomass (Mg ha^−1^)	3.75	0.95	12.05	2.80	4.70
Carbon (Mg ha^−1^)	1.66	0.42	12.05	1.24	2.08

S_ӯ_t: absolute sample error; (S_ӯ_t) %: relative sample error; α: 0.05

When quantifying the dry biomass stocks stored in the litter of a typical Brazilian savannah in the sub-basin of the São Francisco River, in the north of Minas Gerais State, Morais [22] found higher values, on mean, 6.24 Mg ha^−1^ of dry biomass stored in the litter, which corresponds to a mean stock of carbon of 3.05 Mg ha^−1^. But, among the five fragments studied by Morais [22], one presented values very close to this study, with means values of dry biomass and carbon present in the litter of 3.17 and 1.04 Mg ha^−1^, respectively.

### Biomass and carbon total stocks

The total aboveground biomass of the study area was 12.88 Mg ha^−1^, corresponding to the total stock of 5.91 Mg ha^−1^ of carbon. The biomass estimation methodologies are very varied in the existing studies; few have tried to quantify the stocks in the different compartments. In studies that considered total aboveground biomass (arboreal, herbaceous-shrub and litter compartments) in typical Brazilian savannah values ranged from 12.55 to 76.63 Mg ha^−1^ [1, 7, 27, 33].

According to Castro and Kauffman [7], the total aboveground biomass (including trees, herbaceous-shrub layer and litter) tends to be larger as vegetation tends to be denser, and found that biomass increased along the gradient of 5.5 Mg ha^−1^ in the napeadic grassland (“campo limpo”) for 29.4 Mg ha^−1^ in dense savannah. The values presented in this study are within this limit of variation, tending to the lower limit, since the OBS has more open vegetation, with spaced and lower trees, which will reflect less biomass per area.

**Fig. 2:**
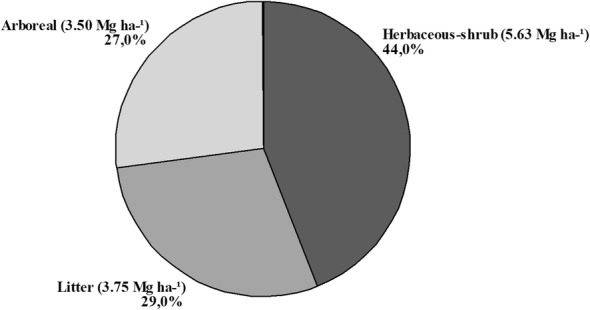
The distribution of the total aboveground biomass stock per compartment evaluated in the OBS

The largest stock of total aboveground biomass is in the herbaceous-shrub component, which represents 44.13% of the total, of which 42% are grasses (Fig. 2). The lowest stock is in the arboreal compartment, representing 27% of the total (Fig. 2).

### Environmental fire considerations

The highest biomass stock in the herbaceous-shrub compartment of the open Brazilian savannah shows a high fragility of this environment about the fire. Because the predominance of this gramineous stratum in this environment of dry climate with a high risk of burning, when under the effect of fire, burns easily, releasing carbon which changes the nutritional dynamics of the ecosystem [42]. According to Pivello and Coutinho [30], on mean, 95% of the nitrogen and between 42 and 59% of the phosphorus, potassium, calcium, magnesium, and sulfur contained in the biomass of the herbaceous-shrub vegetation of Savannah are released into the atmosphere after the burn, requiring a least interval of 3 years between successive fires to promote cycling without affecting the nutritional balance of the ecosystem.

The litter, responsible for 29% of the biomass stock in the OBS, is also a sensitive material to the fire activity in these natural open environments, especially in the sandy soil areas. Castro and Kauffman [7] observed that litter combustion efficiency is higher in open environments where solar radiation is higher. According to Miranda and Sato [20], the presence of trees and shrubs in the more closed savannah formations influence the local microclimate, changing the characteristics of the fire.

The high frequency of burning in these environments reduces the recruitment of arboreal individuals, favoring the establishment of herbaceous vegetation by reducing the density of trees in the ecosystem [23]. Thus, the indiscriminate action of fire over time can change the dynamics of the tree compartment, decreasing the diversity and number of trees in the ecosystem. Furthermore, the negative impacts on the arboreal compartment in the savannah caused by the indiscriminate fire effect and unsustainable management also reflect the biomass and carbon stocks of the roots and other subterranean organisms, since in this case there is a substitution of a complex and deep root system to another homogeneous and superficial. Concurrent to the reduction of the density of arboreal individuals, there is also the change in the litter stock, due to its strong relationship with the presence of the arboreal layer. Werneck et al. [41], observed that the quantitative differences in litter production in three parts of a deciduous forest occurred due to the structural differences and consequent formation of a more developed canopy.

The litter comprises the compartment responsible for the connection between the aerial and underground matrices, behaving as a largesource of nutrients and carbon in the soil, usually dystrophic in Cerrado biome [16]. It is also known that inadequate management of arboreal vegetation can lead to soil degradation, and more in environments such as these, where soils with high levels of sand and naturally fragile, predominate [40]. Thus, the main carbon drain in the biome, the soil, can be highly altered, whose conservation, as well as the carbon storage capacity, is related to the presence of the arboreal vegetation, occurring, according to Aduan et al. [2], stocks under the trees.

When evaluating the dynamics of the arboreal compartment in typical savannah areas sampled in the Cerrado biome, Miranda [21] observed that areas sampled in conservation units showed differences in the stocks between measurements. In an area with frequent fire, the biomass decreased over time, while in another area that does not experience the fire, there was an increase in biomass, corroborating the fact that the indiscriminate fire changes the structure and stocks in Savannah [21]. Areas where fires occur at least intervals of 5 years, biomass has increased over time. The mean rate of increase in carbon was 0.13 t ha^−1^ year^−1^ [21].

According to Bustamante and Oliveira [6], floristic and structural changes of the vegetation caused by fire, concomitant with the increase of the herbaceous-gramineous vegetation, increase the risk of new fires, besides reducing the albedo and the roughness of the vegetation. These changes are, on a larger scale, responsible for increases in temperature and flows of chemical elements to the atmosphere and reductions in rainfall, which in turn increase the chances of new fires are occurring. According to Hoffman and Jackson [17], physical changes in savannah vegetation can reduce evapotranspiration and heat flow into the atmosphere, reducing rainfall and increasing the frequency and intensity of summer. Thus, the intense anthropic pressure added to the natural fragility of the sandy soils of this region and to the particularity of the environmental attributes of this phytophysiognomy, point out the immediate necessity of adopting conservationist measures that promote the sustainable use of these environments, avoiding the indiscriminate conversion of the areas of remaining natural vegetation, causing high environmental damage.

## Conclusions

Based on the precision and residual distribution measures, the Schumacher-Hall (non-logarithmic) equation presented better results to estimate the individual biomass of OBS trees. Total aboveground biomass considering all compartments above the ground was 12.88 Mg ha^−1^, corresponding to a total stock of 5.91 Mg ha^−1^ of carbon. The arboreal compartment represents the smallest part of the stocks, and the grasses predominate storing a large amount of biomass and carbon.

The observed values are in the interval verified for other areas OBS, showing that the values verified for savannah influenced by the sandstones (Urucuia Formation), under Quartzipsamments and Oxisols, are at the lower limit of this range, due to the nutrient-poor nature of these classes of soils.

The distribution of stocks in the different compartments above the ground points to the fragility of this environment to the random fire effect, common in the region. That shows the need for conservation measures for vegetation maintenance and soil protection to preserve adequate nutrient cycling in the ecosystem.

## Abbreviations

OBS: open Brazilian savannah
D_30cm_: diameter at 30 cm from the ground level
D_1.30m_: diameter at 1.30 m from the ground level
IVI: importance value index
h: total height
R^2^_adjust._: adjusted coefficient of determination
Sxy%: standard error of estimate in percentage terms
